# Influence of Spacer Design and Module Geometry on the Filtration Performance during Skim Milk Microfiltration with Flat Sheet and Spiral-Wound Membranes

**DOI:** 10.3390/membranes10040057

**Published:** 2020-03-26

**Authors:** Martin Hartinger, Jonas Napiwotzki, Eva-Maria Schmid, Dominik Hoffmann, Franziska Kurz, Ulrich Kulozik

**Affiliations:** Chair of Food and Bioprocess Engineering, Technical University of Munich, 85354 Freising, Germany

**Keywords:** skim milk, SWM, parallel spacer, module architecture, deposit layer control, flat sheet test cell

## Abstract

Spacer design in spiral-wound membranes (SWMs) significantly affects the axial pressure drop in the flow channel but also the deposit layer removal. However, the effects of the spacer design and feed flow distribution in the module on the filtration performance have not yet been investigated during the highly fouling-susceptible fractionation of proteins from skim milk by SWMs. Therefore, a parallel spacer with no turbulence promotion and a less homogeneous feed flow distribution in the SWM was compared to a diamond spacer with regard to its impact on deposit formation and filtration performance. The experiments were conducted in a flat sheet test cell and in SWMs. The parallel spacer induced a more homogeneous deposit layer formation. However, no difference in filtration performance could be observed in the experiments with the test cell. Even though deposit layer formation dominates the microfiltration, its amount and spatial distribution could not be directly linked to the filtration performance. Furthermore, both spacers were assessed in SWM. Despite the higher crossflow velocity applicable in the more open channels of the parallel spacer, the performance of the parallel spacer was inferior to the diamond spacer. This was independent of the viscosity of the feed. Due to the high curvature of the membrane sheets close to the permeate collection tube, the cross-section of the flow channels in the SWM equipped with the parallel spacer was reduced. This resulted in a distinctly lower deposit layer control and performance, which could not be compensated by the resulting higher crossflow velocity far from the permeate collection tube.

## 1. Introduction

Deposit layer formation is one of the major obstacles in the application of membrane technology, since filtration performance is significantly affected by the fouling layer on the membrane surface. Thus, a number of works have investigated the causes and mechanisms of deposition [1,2,3,4]. In the case of membrane technology in food manufacture applications, proteins are often the major foulant [5]. Proteins, such as micellar caseins, can form strongly compressible deposits [6]. Compression of the deposit causes a distinct reduction of the permeability of the filtration system, as the deposit layer acts as a secondary filtration layer and dominates the filtration process. According to Davis and Leighton [7], wall shear stress is the major factor for deposit removal from the membrane surface. Increasing the crossflow velocity results in a higher wall shear stress and a more effective deposit layer control. However, as it was shown by von der Schulenburg et al. [8], the feed velocity is spatially distributed in the spiral-wound membrane (SWM) in the radial direction and so is the filtration performance. The hypothesis is that a more homogeneous distribution of the feed velocity in the module induces better deposit layer control and enhances filtration performance.

Since spacers define the flow channel in SWM and the local flow regime in close vicinity of the spacer strands, their effect on the homogeneity of the feed flow distribution and the resulting filtration performance should be investigated. It is known that the spacer causes an axial pressure drop, which increases disproportionally with the crossflow velocity [9]. The pressure drop but also the filtration performance were reported to be strongly dependent on the geometry of the spacer [10,11,12,13]. Da Costa et al. [10] described that the axial pressure drop in spacer-filled channels is caused by four additive factors: Viscous drag on the spacer, viscous drag on the channel walls, form drag, and kinetic losses due to changes in the flow direction. All factors except for the viscous drag on the channel walls are dependent on the spacer geometry but also on the viscosity of the feed. Thus, a dependency of the pressure drop on the interaction between the spacer geometry and feed viscosity is to be expected.

This is especially relevant for the fractionation of skim milk by microfiltration (MF). In this process, the skim milk feed needs to be concentrated to obtain the optimum fractionation performance with regard to the feed composition [14]. This severely increases feed viscosity and the feed becomes increasingly shear thinning [15]. Thus, the optimum feed spacer design for milk protein fractionation by MF is dependent on the feed viscosity and should be investigated.

Apart from the influence of the spacer design on the pressure drop, the spacer geometry also affects the flow pattern in the SWM. It was shown that the spacer geometry can cause eddies, i.e., an inhomogeneous wall shear stress distribution in spacer-filled channels [16]. Thus, deposit layer formation is spatially distributed in the spacer net as well. During ground water filtration, deposit layer formation predominately takes place close to the contact area of the diamond spacer and the membrane, where wall shear stress is low [17]. Furthermore, it was shown by computational fluid dynamics simulation (CFD) that different spacer geometries result in different concentration profiles in the flow channel [18]. In this regard, deposit layer formation and flux can be influenced distinctly by the spacer geometry. Arunkumar et al. [12] observed that an open channel caused a lower flux and a lower pressure drop during ultrafiltration (UF) of an antibody solution compared to a diamond-shaped spacer.

Since the deposit layer acts as a secondary filtration layer on the membrane, the amount of deposited protein and thus, the geometry of the spacer should not only affect the flux but also protein permeation. The amount of deposited protein is referred to as the quantity of protein, which is bond to the membrane surface and can be detected by a method described previously [19].

Our goal was to assess the deposit formation and filtration performance of a standard diamond spacer and a parallel spacer during skim milk MF. On the one hand, the influence of the form drag [10] on the pressure drop is lower with the parallel spacer but on the other hand the geometry does not promote turbulence but behaves similarly to a tube. The influence of the spacer geometry on the filtration should be assessed in a flat sheet application. Apart from that, the influence of the spacer design in the application of SWMs at varying feed concentration factors (CFs) should be addressed in order to assess the influence of the feed volume flow distribution in the module and the feed viscosity on the filtration performance.

## 2. Materials and Methods

### 2.1. Crossflow Filtration Rigs and Experimental Procedure

The experiments were conducted on a filtration test cell and a pilot-scale filtration plant for SWMs. The 0.1-µm polyvinylidene fluoride (PVDF) membrane (V0.1, Synder Filtration, Vacaville, CA, USA) was either used as a flat sheet or manufactured to a SWM using the same membrane material and the same spacer design in both cases.

#### 2.1.1. Flat Sheet Test Cell and Experimental Design

**Figure 1:**
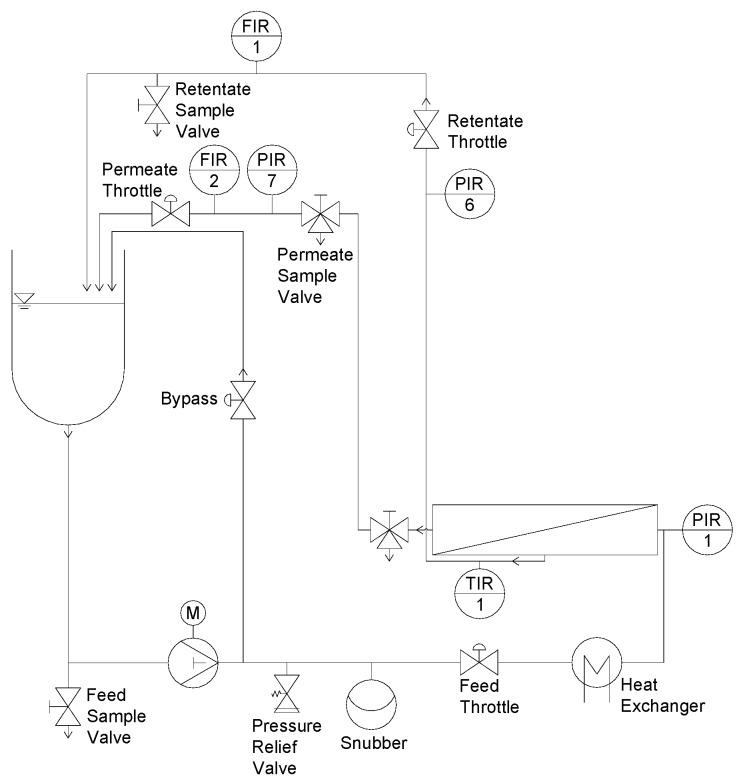
Test cell filtration plant (adapted from [9]).

Details on the test cell filtration rig, the geometry of the filtration unit, and the experimental design are described elsewhere [9]. In brief, filtration experiments were conducted on a flat sheet test cell (SIMA-tec, Schwalmtal, Germany) as shown in Figure 1. The unit consists of a receiver tank (3 L), a piston pump, a snubber to create a continuous flow, a heat exchanger for feed tempering, and the filtration unit. Like in a SWM, the flow channel height was determined by the spacer height.

Prior to filtration, a new flat sheet membrane was soaked in deionized water for 24 h at 4 °C. The membrane was inserted into the test cell with the selective layer pointing downwards and then conditioned (20 min, 50 °C, 0.4% vol/vol Ultrasil 69; Ecolab Deutschland GmbH, Monheim am Rhein, Germany). A volume of 2.5 L of UF milk concentrate (CF 3) was heated to 25 °C and sheared for 30 min to restore the viscosity directly after the concentration. Afterwards, the concentrate was cooled to the filtration temperature of 10 °C. In order to flush the filtration plant, 0.5 L of concentrate were drained to remove the mixed phase before retentate and permeate were recirculated. The axial pressure drop (Δp_L_) of the plant was set to 1.0 bar m^−1^ and the Δp_TM_ was adjusted. The axial pressure drop resulted in a mean crossflow velocity of 0.21 and 0.14 m s^−1^ (0.26 and 0.19 m s^−1^ considering the reduction of the flow channel by the presence of the spacer) for the diamond and the parallel spacer, respectively. The mean crossflow velocity v was calculated according to Equation (1) with the feed volume flow $\dot{V}$ and the free cross-section of the feed channel A with a height of 46 mil (1.17 mm) without the spacer:

(1) $$v=\frac{\dot{V}}{A}$$

After 60 min, permeate and retentate samples were taken and the filtration was terminated. This time was sufficient to reach steady state filtration as we showed in an earlier work [20]. The membrane was removed from the test cell and immersed in deionized water (20 °C) for 30 s to remove residual milk loosely adhered to the deposited protein before further analyses. The pressure drop measurements were conducted with water at 10 °C without permeate production.

#### 2.1.2. Pilot-Scale Filtration Plant for SWM and Experimental Design

**Figure 2:**
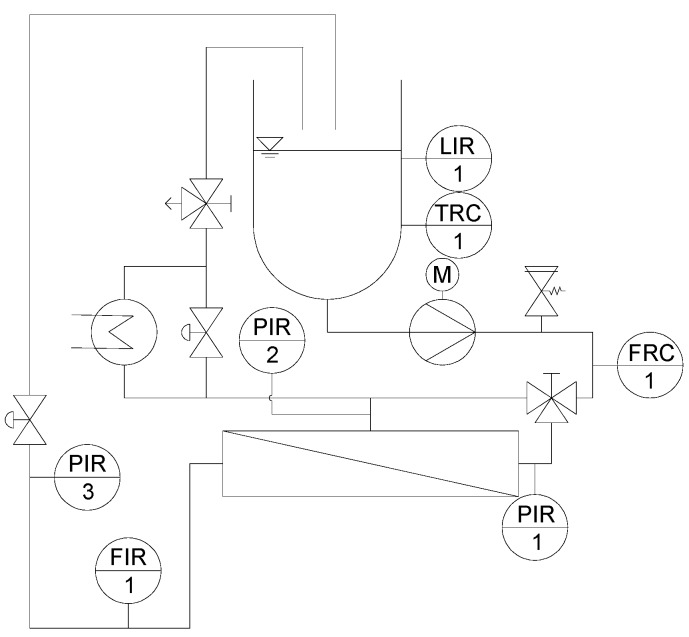
P&I diagram of the pilot scale plant for SWM (LIR: Level indicator recorder; TRC: Temperature recorder controller; PIR: Pressure indicator recorder; FRC: Flow recorder controller; FIR: Flow indicator recorder) [20].

Experiments on SWM were conducted on a pilot-scale filtration plant, which is described in detail elsewhere [20]. The piping and instrumentation (P&I) diagram of the plant is shown in Figure 2. A multistage centrifugal pump created the crossflow to achieve 1.0 bar m^−1^ axial pressure drop in the membrane module.

The experimental design is described in detail by Hartinger and Kulozik [14]. The feed (skim milk) was used at CF 1, 2, or 3. The concentration factor was calculated by Equation (2) with the concentration of casein in the concentrate c_conc._ and in skim milk c_skim milk_:

(2) $$\mathrm{CF}=\frac{c_{\mathrm{conc}.}}{c_{\mathrm{skim}\;\mathrm{milk}}}.$$

In experiments with higher CF, the feed was concentrated by MF at 0.5 bar transmembrane pressure. After the CF value was reached, the permeate was recirculated and the transmembrane pressure was increased every 30 min by 0.2 bar up to a maximum of 1.5 bar.

Table 1 sums up the geometrical properties of the SWM and the test cell.

### 2.2. Filtration Fluid Skim Milk and Calculations

Pasteurized skim milk (74 °C; 28 s) was obtained from a local dairy (Molkerei Weihenstephan, Freising, Germany). For experiments on the pilot plant, skim milk was used and if necessary concentrated in a first processing step by MF as described above. Due to the low active membrane area, filtration experiments on the test cell were conducted with skim milk concentrated to CF 3 by UF (10 °C; 10 kDa PES membrane, Pall GmbH, Dreieich, Germany) prior to the experiment. This milk UF-concentrate contained a three-fold amount of proteins (approximately 120 g L^−1^), whereas the composition of all other solutes was equal to milk at CF 1. The composition of the major protein fractions can be found in Table 2. It has to be noted that the way of concentration to CF 3 (either by UF or by MF) did not affect the filtration performance [14].

The permeation of whey proteins was assessed by determination of its main component β-lg. Permeation P was calculated by the concentration of β-lg in the retentate c_r_ and in the permeate c_p_ according to Equation (3):

(3) $$P=\frac{c_{p}}{c_{r}}\times 100\;\%.$$

### 2.3. Spacer Design

**Figure 3:**
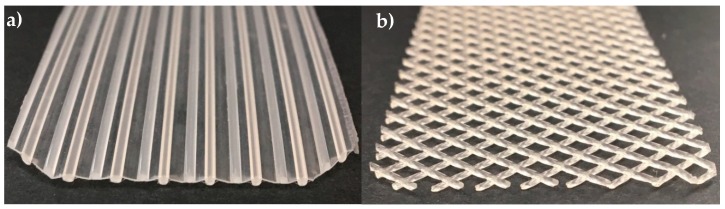
Geometry of the parallel (**a**) and the diamond spacer (**b**).

In this study, the influence of the spacer design on the spatial distribution of the deposit layer, the crossflow velocity distribution in the SWM, and the filtration performance at varying CF values was investigated. Two spacer geometries were tested: a parallel and a diamond-shaped spacer, both with a height of 46 mil (milli-inch) or 1.17 mm. The parallel spacer (Figure 3a) was made available by Synder Filtration (Vacaville, CA, USA). The parallel spacer held ribs in an almost semicircular shape (diameter 0.65 mm) in axial direction, with a distance of 4.9 mm from one another on both sides of a backbone (thickness 0.25 mm). It has to be noted that the backbone is slightly corrugated due to the manufacturing process. The spacer shape resulted in a maximum feed channel height of 0.65 mm. The spacer had a void volume fraction of 0.73. The void volume fraction ε was calculated according to Equation (4) with the total volume of the channel V_tot_ and the volume of the spacer V_spacer_ [10]:

(4) $$\varepsilon =\frac{V_{tot}-V_{spacer}}{V_{tot}}.$$

It has to be noted that the backbone divided the feed channel in two sections. Excluding the backbone of the spacer, the void volume fraction was 0.93.

The diamond spacer was manufactured by Intermas (Barcelona, Spain) (Figure 3b). It is a non-woven spacer with two layers of almost circular-shaped spacer strands spliced together. The stands were positioned orthogonally to each other. The distance between two strands was 3.6 mm. At the junctions between the two spacer strand layers, the thickness of the spacer strand was 0.94 mm. Between the junctions, the strand thickness was 0.65 mm. It has to be noted that the strand geometry was not perfectly spherical but slightly elliptic with slight deformations. The spacer had a void volume fraction of 0.82.

### 2.4. Quantification of Deposited Protein on Membranes

The quantification of proteinaceous deposit layers was done by RP-HPLC. To do so, the deposit layer on part of the membrane (area of 20 cm^2^) was completely dissolved in 5 mL of a guanidine buffer (6 M guanidine hydrochloride, 21.5 mM trisodium citrate, 19.5 mM dithiotreitol in a 0.1 M bis tris buffer) described by Dumpler et al. [21] at 20 °C for 60 min. This time was sufficient to completely remove all the proteins from the deposit layer. The dissolution kinetics of the deposit layer into the buffer can be found in the Supplementary Materials (Figure S1). The supernatant was then injected into the RP-HPLC without further treatment and analyzed according to Dumpler et al. [21]. With this method, a determination of the mean deposition of protein on the membrane surface was possible. However, an extraction of the deposit layer on a small scale as required for the analysis of the local effects of the spacer design was not possible. Therefore, a spatially resolved semi-quantitative method by means of staining was used additionally to the quantification by means of RP-HPLC.

### 2.5. Membrane Staining and Image Evaluation

The method used for staining and image evaluation is described in detail elsewhere [19]. In brief, the membrane was stained for 10 min by a Coomassie Brilliant Blue (CBB) solution and destained in ethanol for 3 min. After drying, the membrane was analyzed by a Gel Doc XR+ (Bio-Rad Laboratories, Inc., Hercules, CA, USA). It took a black-and-white image (width 1392 pixel, height 1040 pixel) of the membranes (exposure time 0.6 s) with a charge-coupled device (CCD) camera. A virgin membrane stained with Coomassie Brilliant Blue served as a blank with a gray value of 1251.5. To ensure that the illumination was equal for all measurements, a reference (gray plastic plate) was additionally measured and evaluated in each measurement. Since the gray value of the reference would change, a variation in illumination could be detected.

As the blue color of the stained proteins was transformed into a black-and-white image, darker areas mark a higher protein concentration and a more intense deposition. The gray value of the images was evaluated by ImageJ (Version 1.51f, National Institutes of Health, Bethesda, MD, USA). The calibration of the staining method can be found in Hartinger et al. [19].

#### 2.5.1. Visualization of the Deposit Layer Distribution in a False Color and a Topographic Image

A spatially resolved analysis of the deposit layer formation was performed. Using ImageJ, the gray values were correlated to a color spectrum. The spectrum referred to the correlation function depicting the deposit layer´s spatial distribution. The lookup table (LUT) can be found elsewhere [19]. In a further step, the gray values were illustrated topographically. The description can be found in [19] as well. The minimum and maximum picture brightness of the 16-bit image was set to 0 and 4095 and the LUT was applied. Following this, the plugin Interactive 3D Surface Plot was used (grid size 1024, smoothing 8, perspective 0, lighting 0.15, scale 1.5, z-scale 0.23, min 0%, max 100%, x-angle 54°, z-angle-64°) to create topographic images of the deposition.

#### 2.5.2. Position-Resolved Evaluation of the Deposited Protein

**Figure 4:**
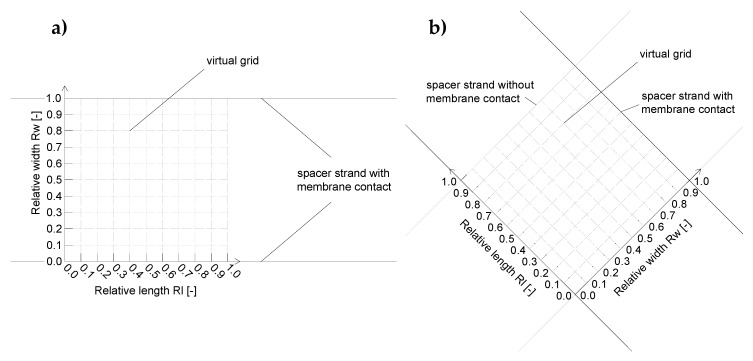
Analysis pattern on the membrane surface for the parallel spacer (**a**) and the diamond spacer (**b**). The 10 × 10 elements virtual grid was used to analyze the deposition intensities spatially resolved in the various positions between the spacer strands and ribs, respectively.

Since false color and topographic images are hard to compare in terms of deposit layer formation, the gray value and thus the amount of deposit layer were evaluated parallel and orthogonally to the spacer strands. A pattern of parallel lines describes the interspace between the spacer strands (Figure 4).

The gray values were transformed into the amount of deposited protein by a correlation function. As a statement of place, the relative length (Rl) and relative width (Rw) were used. A relative length or relative width of 1 refers to the distance between the centers of two spacer strands or ribs in the direction parallel and perpendicular to the spacer strand or ribs, respectively. Note that the absolute lengths of the relative length units differ between the spacer designs.

### 2.6. Data Plotting and Fitting

Data was plotted using OriginPro 2017G (OriginLab Corporation, Northampton, MA, USA). The program also fitted the data with an exponential function using a Levenberg Marquardt as an iteration algorithm or via b-spline. Error bars show the standard deviations of two individual experiments. The plots of the graphical analysis are a single determination.

## 3. Results and Discussion

### 3.1. Deposit Layer Formation Caused by a Parallel Spacer

Deposit layer formation is known to reduce the filtration performance of membranes tremendously. Therefore, we investigated the influence of the spacer geometry on the spatial distribution of protein deposition on the membrane surface during skim milk filtration. The deposit layer mainly consisted of casein (compare Supplementary Material Figure S2) as already shown by Jimenez-Lopez et al. [22] during ceramic MF of skim milk (0.1 µm).

**Figure 5:**
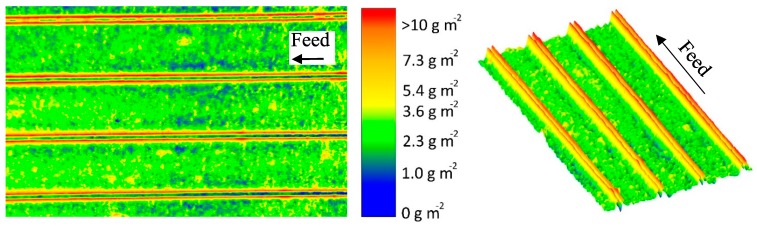
False color and topographic image of the deposit layer formed by a parallel spacer after filtration of concentrated skim milk (CF 3, Δp_L_ = 1.0 bar m^−1^, mean crossflow velocity of 0.14 m s^−1^, Δp_TM_ = 0.5 bar). The average amount of deposited protein determined by HPLC was 4.9 g m^−2^. The membrane after staining can be found in the Supplementary Material (Figure S3).

Figure 5 shows a false color and a topographic image of the distribution of protein deposited on the membrane surface after the MF of skim milk with CF 3 using a 46 mil parallel spacer. The local concentrations (determined via the gray values and calibration curve) of the stained deposits were converted to a false color. Red areas mark a protein content of >10 g m^−1^, beyond which no further differentiation of the amount of protein is possible. The cumulative distribution can be found in the Supplementary Material (Figure S5). It can be seen in Figure 5 that the deposit layer´s pattern fits the geometry of the spacer.

In the areas in which the spacer is in direct contact with the membrane, the deposit layer is predominantly low. As no protein was detected to be remaining on the spacer after removal from the membrane, this indicates that no filtration took place below the spacer ribs. Otherwise, protein deposition would be expected to be intense, since particle-removing forces are low due to a lack of crossflow below the spacer ribs. Nevertheless, certain areas in the membrane-spacer contact zone are fouled with up to 10 g m^−2^. These areas are randomly distributed along the membrane-spacer contact zone. This allows two conclusions: Firstly, no full contact between the membrane and spacer exists. Thus, parts of the membrane in the contact zone close to the spacer contribute to the filtration. Due to the low crossflow velocities in the contact zone, flux and protein permeation are expected to be rather low compared to the rest of the membrane. Additionally, excessive mixing between the branch currents streaming in the gap between the spacer ribs is not to be expected. Secondly, a removal of the spacer from the membrane prior to staining does not detach parts of the deposit layer. Otherwise, sharp edges would be expected. Hence, the deposition patterns shown in Figure 5 correspond to the state during filtration. Therefore, in most areas in which the spacer rests on the membrane, no filtration takes place.

The effect of the spacer can also be observed close to the contact zone between spacer rib and membrane. More than 10 g m^−2^ of protein are deposited, since a boundary layer forms. The crossflow velocity is reduced due to frictional energy losses caused by the spacer. Thus, the wall shear stress decreases toward the spacer, causing a more intense particle deposition.

**Figure 6:**
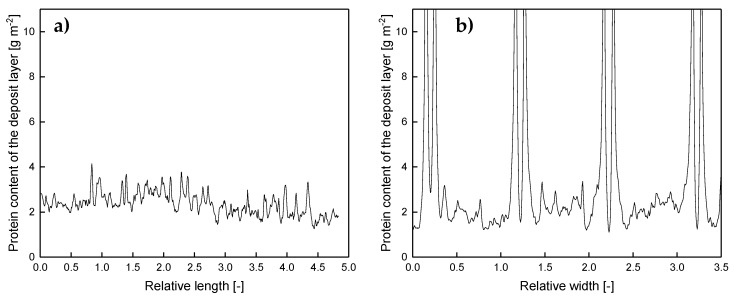
Deposition caused by a parallel spacer parallel (at Rw of 0.5) (**a**) and orthogonally (**b**) to the flow lines of the feed. Rl and Rw refer to the distance between two spacer ribs and correspond to 4.9 mm. Graphs show data obtained from a single experiment. Values above 10 g m^−2^ could only be determined qualitatively and are therefore not shown.

Except for the areas close to the spacer, the deposit layer is homogeneously distributed all over the membrane with about 2 to 4 g m^−2^. Due to the lack of baffles, the flow is undisturbed. Wall shear stress values are expected to be constant along the flow lines parallel to the ribs, causing a homogeneous deposition. This can be concluded from Figure 6a, which shows the deposit layer distribution as a function of the relative length according to the spatial grid defined by Figure 4a. Hence, it can be inferred from the deposit layer pattern that no small-scale variations in flux and protein permeation prevail with the parallel spacer except for the areas in the direct vicinity of the spacer membrane contact zone. The slight deviations in deposited protein are presumed to be due to inhomogeneities in the selective layer´s properties. Thus, the amount of deposited protein varies to a certain extent even though the conditions can be assumed to be the constant.

Contrary to the observations along the relative length axis, inhomogeneities in the deposit layer formation perpendicular to the feed flow can be observed (Figure 6b). Close to the ribs, the deposition increases steeply. Outside of the boundary areas, the deposition stays constantly at a lower value. The size of the boundary layer, where the accumulation of protein deposit takes place, could be estimated by measuring the distance between the maximum concentration of deposited protein (defining the position directly at the spacer rib) and the point, at which the amount of deposited protein reached the level of the freely overflown areas (far from the spacer ribs). The size of the boundary layer was roughly 0.089 ± 0.040 Rw, which corresponds to 0.044 ± 0.019 mm. Due to the crossflow velocity and wall shear stress being not fully developed close to the ribs, friction effects reduce the erosive forces and enhance deposition.

It was found that the deposit layer pattern of the parallel spacer is homogeneous along the flow path except for areas close to the spacer contact zone. This is due to the lack of flow restrictions and the lack of eddies. In turn, the crossflow velocity and wall shear stress can be assumed to be practically constant in the areas of low fouling. Furthermore, we assume that friction causes lower crossflow velocities close to the spacer. This increases the deposition of proteins since wall shear stress values are lower compared to the areas of undisturbed flow. Therefore, we can confirm that the local flow regime directly affects deposition.

### 3.2. Deposit Layer Formation Caused by a Diamond-Shaped Spacer

**Figure 7:**
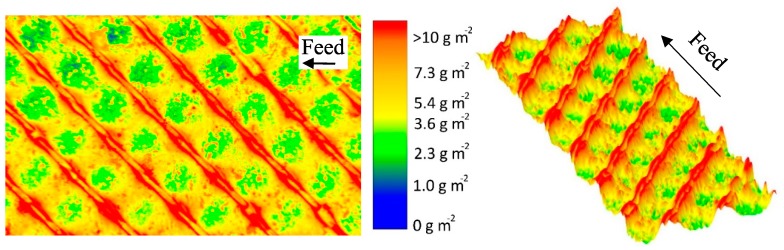
False color and topographic image of the deposit layer formed by a diamond-shaped spacer after filtration of concentrated skim milk (CF 3, Δp_L_ = 1.0 bar m^−1^, Δp_TM_ = 0.5 bar). The average amount of deposited protein determined by HPLC was 5.6 g m^−2^. The membrane after staining can be found in the Supplementary Material (Figure S4).

Comparable to the approach for the parallel spacer, we analyzed the deposit layer distribution caused by the diamond spacer in the test cell. The results on the parallel spacer show the influence of the flow regime on the deposition of proteins. This can also be expected from the diamond spacer as it causes eddies in the feed flow on a small scale. In this study, we investigated a non-woven spacer with two layers of spacer strands orthogonal to one another. Only one layer of spacer strands is in contact with one membrane surface and spatial crossflow velocities and wall shear stresses differ strongly in the spacer net. The protein deposition also changes along the flow path, which can be seen in Figure 7. The protein accumulation is most pronounced behind the spacer strand with membrane contact. This is due to the low crossflow velocities directly behind the spacer strand. This was also observed in computational fluid dynamics (CFD) simulations [23]. Based on the findings of Grosse-Gorgemann et al. [24] and Fiebig [25] for heat transfer, Li et al. [26] stated that slowly rotating transversal vortices form behind spacer strands at Reynolds numbers (based on the channel height) below 100. The conclusion is that due to the vortices, the mass exchange with the bulk phase is reduced. Thus, a concentration and an increased deposition close to the spacer strands takes place. Since we found Reynolds numbers below 100 in our studies, the formation of vortices behind the spacer strands might take place. Apart from that, a low wall shear stress and thus low removal forces occur behind the spacer strand due to the low crossflow velocity.

Since the wall shear stress is also low in front of the spacer strand with membrane contact, much protein is deposited in this area as well. In the membrane–spacer contact zone, much lower amounts of proteins are deposited, as already shown with the parallel spacer, due to the lack of filtration.

Apart from those areas adjacent to or covered by the spacers, the deposit layer distribution on the membrane in the area between the spacer strands is also inhomogeneous. In the areas close to the spacer without membrane contact, deposit layer formation was found to be lower than average (5.6 g m^−2^ determined by HPLC). Due to a narrowing of the flow channel, the crossflow velocity (mean value of 0.21 m s^−1^) increases. In turn, the wall shear stress is increased, reducing deposit layer formation. Koutsou et al. [16] calculated the spatially resolved wall shear stress on the membrane covered with a diamond-shaped spacer (hydrodynamic angle 90° (angle between two spacer strands facing the feed flow direction)). They also reported higher wall shear stress values in the areas of flow constriction. Additionally, the authors reported an increased mass transfer coefficient. Concentration polarization is less pronounced in these areas and deposit layer formation is less likely to happen [27].

**Figure 8:**
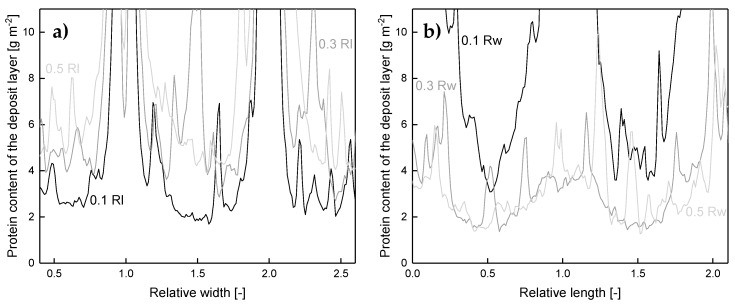
Deposited protein on the membrane surface after filtration of concentrated skim milk (CF 3) as a function of the axial position on lines parallel to the spacer strands without (**a**) and with (**b**) membrane contact in varying distance. One Rl/Rw refers to the distance between the centers of two adjoining spacer strands and corresponds to 3.5 mm. The Rl and Rw of 0, 1, and 2 are at the middle of the spacer strand. Graphs show data obtained from a single experiment. Values above 10 g m^−2^ could only be determined qualitatively and are therefore not shown.

The results show that deposit layer formation is directly affected by the spacer geometry due to the effect on the feed flow. Most likely, spatially distributed alterations of the wall shear stress are the determining factor. Behind spacer strands with membrane contact, protein deposition is homogenous and pronounced. With further distance from the spacer strand in the flow direction, an increasing wall shear stress causes the degradation of deposited proteins. In front of these strands, protein deposition is pronounced in the areas without flow constriction. Furthermore, it was observed that the spacer without membrane contact causes an increase of the local crossflow velocity, resulting in a higher wall shear stress and less deposition. The extent of inhomogeneities on the membrane can be seen in Figure 8. It plots the deposit layer distribution as a function the axial position on lines parallel to the spacer strands without (a) and with (b) membrane contact. For the sake of clarity, only three lines are shown: 0.1, 0.3, and 0.5 Rl and Rw, respectively, behind the spacer membrane contact.

It can be seen that a two-dimensional pattern is generated by the spacer. For a position with a relative width (Rw) of 0.5 (Figure 8a), the amount of deposited protein varies between 4 g m^-2^ and more than 10 g m^−2^. Since the spacer strands without membrane contact narrow the flow channel, the crossflow velocity and wall shear stress are enhanced locally as reported above. This results in a more intense removal of deposited protein and a deposition pattern closer to the spacer strand without membrane contact (lower Rl). Thus, the amount of deposited protein decreases to a lowest value of 2 g m^−2^ and the width of the area with a deposition above 10 g m^−2^ decreases. Apart from that, the deposit layer decreases with distance from the spacer strands with membrane contact (Figure 8b). As reported by Kavianipour et al. [23], the crossflow velocity varies due to the spacer strand´s influence on the flow field. Since the feed stream is influenced by the spacer strands, an area of flow separation can occur. This causes a reduction of the local wall shear stress close to the spacer strands with membrane contact and thus more intense fouling. An influence of the spacer without membrane contact is observable independent of Rw. Just the extent of its influence is dependent on Rw.

The results show that the diamond-shaped spacer causes a characteristic deposit layer pattern on the membrane surface directly reflecting the spacer configuration. Thereby, areas with high crossflow velocity and wall shear stress, respectively, are less susceptible to fouling, but at the same time, areas of very poor deposit control exist.

The cumulative fouling distribution of the diamond can be found in the Supplementary Material (Figure S5).

### 3.3. Filtration Performance of the Diamond and the Parallel Spacer

**Figure 9:**
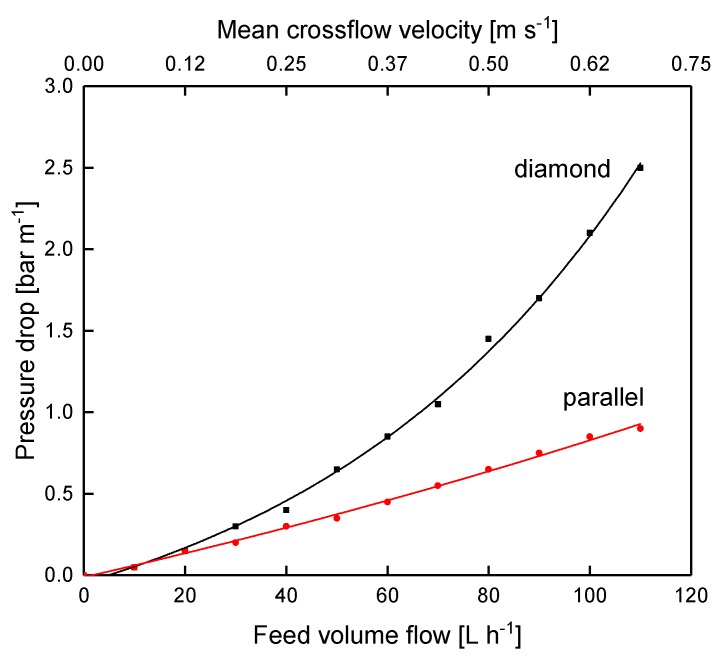
Pressure drop of the parallel and the diamond spacer as a function of the feed volume flow of water at 10 °C without permeate production. The graph shows data obtained from a single experiment.

As reported above, the spacer design significantly affects the deposit layer pattern. As the deposit layer is known to dominate the filtration during skim milk fractionation, we investigated the influence of the spacer design on the filtration performance. For this purpose, we analyzed flux and protein permeation of the parallel and diamond-shaped spacer during skim milk fractionation to obtain a casein and a whey protein fraction in a flat channel test cell. Figure 9 shows the pressure drop of both spacer designs as a function of the crossflow velocity during water filtration.

As expected, the pressure drop increases with increasing crossflow velocity due to enhanced friction. Apart from that, the parallel spacer caused a significantly lower pressure drop. At 80 L h^−1^ (mean crossflow velocity of 0.5 m s^−1^), the pressure drop was 1.45 and 0.65 bar m^−1^ for the diamond and the parallel spacer, respectively. This can also be attributed to frictional losses. As already described by Da Costa et al. [10] and Kavianipour et al. [23], the feed stream changes direction as it flows above and below the spacer strands of the diamond-shaped spacer net. This causes energy dissipation, which is not present with the parallel spacer due to the linear feed flow comparable to the flow in tubular membranes or pipes.

It was shown by Samuelsson et al. [28] that higher crossflow velocities enhance the flux during skim milk MF in the tubular geometry of ceramic membranes. In order to evaluate the filtration performance of two spacers with different geometries, the spacers can be compared either at equal feed volume flows or at equal axial pressure drops. Since the maximum pressure drop in SWMs is a limiting value due to possible module destruction by telescoping, the axial pressure drop is the more relevant value for SWMs and was selected for the comparison. Telescoping describes an axial displacement of the membrane sheets in the direction of the feed flow induced by friction losses along the module, which can cause module destruction.

**Figure 10:**
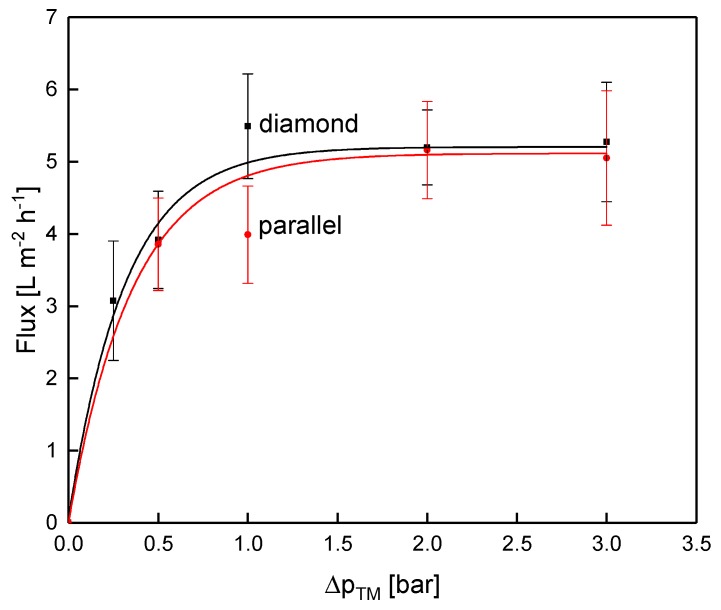
Flux as a function of the transmembrane pressure for the parallel and the diamond spacer during MF of concentrated skim milk (CF 3). Error bars represent the standard deviation of two individual experiments.

The expectation was that the more homogeneous and lower deposition resulting from the parallel spacer leads to a better filtration performance in terms of flux and protein permeation compared to the diamond spacer. Figure 10 shows the flux as a function of the transmembrane pressure during the filtration of concentrated skim milk (CF 3).

With increasing Δp_TM_, the flux increased until the limiting flux was reached at about 5 L m^−2^ h^−1^. Interestingly, and against expectation, the flux did not differ between the parallel and the diamond spacer when operated at the same pressure drop of 1.0 bar m^−1^ even though the feed flow differed distinctly between the two spacer designs. In contrast to the pressure drop in water, the diamond spacer allowed a higher crossflow velocity at CF 3 compared to the parallel spacer (mean crossflow velocity of 0.14 and 0.21 m s^−1^ for the parallel and the diamond spacer, respectively).

**Figure 11:**
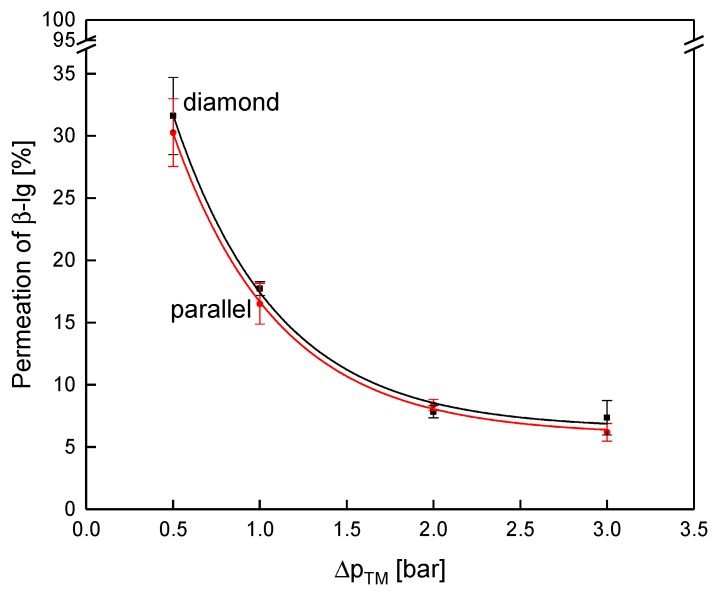
Permeation of β-lg as a function of the transmembrane pressure for the parallel and the diamond spacer during MF of concentrated skim milk (CF 3). Error bars represent the standard deviation of two individual experiments.

Similar to the flux, protein permeation in the test cell was also not dependent on the spacer design (Figure 11).

As expected, the protein permeation decreased with increasing transmembrane pressure due to enhanced deposit layer compression [29]. The permeation of β-lg at 0.5 bar Δp_TM_ was 32% and 30% for the diamond and the parallel spacer, respectively, and decreased to 8% and 8% at 2.0 bar. Again, the spacer design had no decisive influence on the filtration performance when they were operated at the same pressure drop in the flat sheet test cell. Even though the deposit layer formation was distinctly different between the two spacer geometries, no significant effect on the filtration performance was observed in our study. Presumably, the effect of the spacer geometry on the axial pressure drop counterbalances the differences in the mass flow coefficient of both spacer geometries. Since no effect of the spacer design was observed within the test cell, another set of experiments was conducted within SWMs with their curved feed flow channels and more complex architecture.

### 3.4. Filtration Performance of the Diamond and the Parallel Spacer in a Spiral-Wound Membrane at Varying Concentration Factors

The observations on the deposit layer pattern and amount did not go along with the obtained results from the test cell. Apart from that, the curvature of the SWM could not be depicted by the test cell. Therefore, the test cell experiments were also conducted in SWMs at concentration factors of 1 to 3. For the application of spacers, frictional losses in the membrane module have to be traded off against the positive effect of turbulence promotion on the mass transfer. In this regard, a change in viscosity is expected to shift the geometrical optimum of the spacer geometry from a more turbulence-generating shape (at the cost of a greater axial pressure drop) to a less turbulence-generating shape (at the benefit of a lower axial pressure drop).

**Figure 12:**
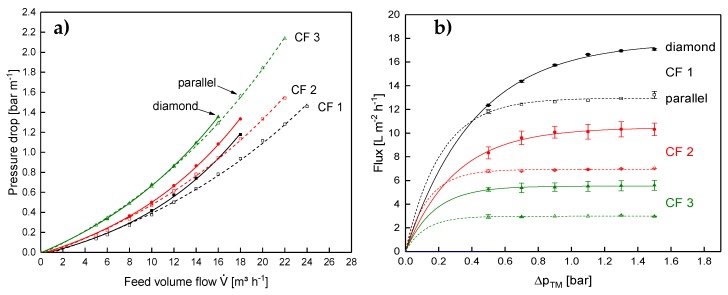
Axial pressure drop as a function of the feed volume flow (**a**) and flux as a function of the transmembrane pressure for the parallel and the diamond spacer in SWM during MF of skim milk at CF 1 to 3. Error bars in (**b**) represent the standard deviation of two individual experiments. Data on the diamond spacer were adapted from Hartinger and Kulozik [14].

The axial pressure drop at varying CF values is shown in Figure 12a for the parallel and the diamond spacer.

The expectation was that the lower amount of flow constrictions would result in a significantly lower pressure drop with the parallel spacer compared to the diamond spacer. This can be observed for low concentration factors. However, the difference between both spacer geometries becomes lower with increasing feed concentration. This means the main positive aspect of the parallel spacer having a lower axial pressure drop and allowing higher crossflow velocities vanishes at high CF values. The difference in behavior between the test cell and the SWM with regard to the axial pressure drop is probably due to the differences in the feed inlet (compare [9]).

In contrast to the results of the test cell, the diamond spacer showed a higher flux in the SWM, not only at low CF but also at higher CF (Figure 12b). This is rather unexpected at first sight, as the crossflow velocity of the parallel spacer was similar to the diamond spacer and not lower like in the test cell.

**Figure 13:**
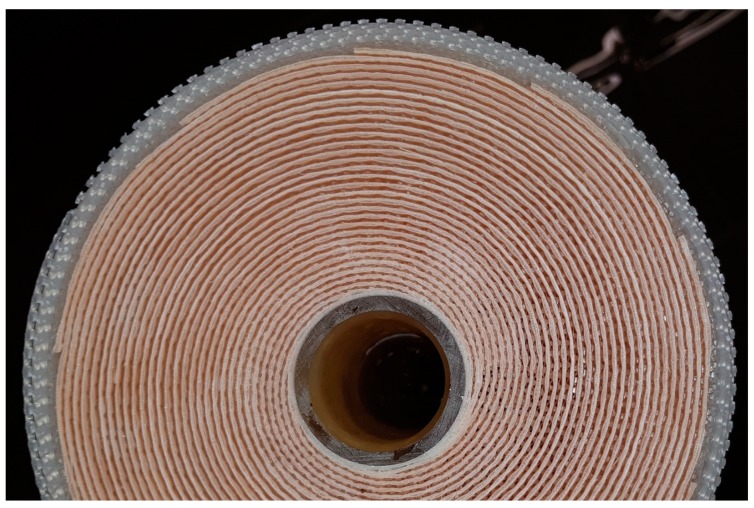
Front face of the SWM with the parallel spacer.

Therefore, the geometry of the flow channels in the SWM and radial distribution of the crossflow velocity have to affect the filtration performance. Figure 13 shows the feed inlet side of the SWM with the parallel spacer.

In the outer part of the module, the membranes and the spacer form a flow path for the feed. In the inner part of the module, however, the spacer is pressed into the membranes. Due to indentation of the spacer into the membrane, the active membrane area decreases. This has already been described by Karabelas et al. [30]. Additionally, hardly any flow channel remains open due to the indentation and the high curvature of the spacer close to the permeate collection tube. In consequence, the open cross-section of the inner channels is lower, resulting in a low crossflow velocity and poor deposit layer control. These effects cannot be observed with the diamond spacer.

**Figure 14:**
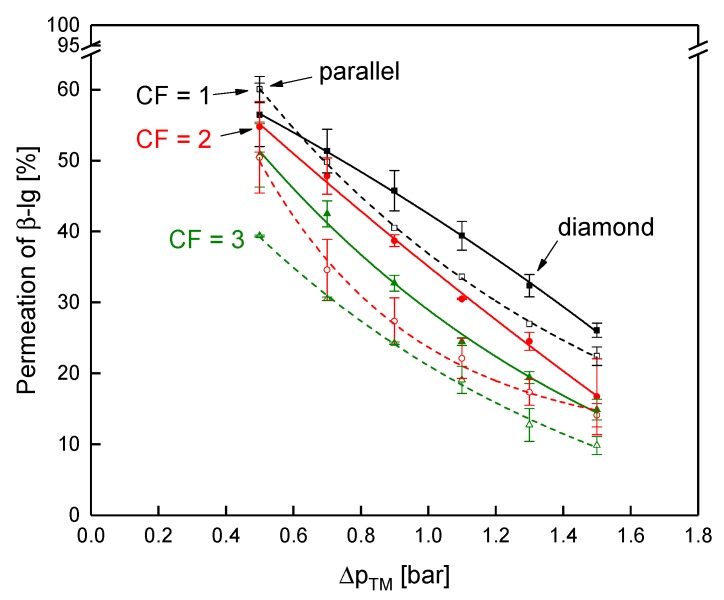
Permeation of β-lg as a function of the transmembrane pressure for the parallel (dashed lines and open symbols) and the diamond spacer (continuous lines and full symbols) during MF of skim milk at CF 1 to 3. Error bars represent the standard deviation of two individual experiments. Data of the diamond spacer was adapted from Hartinger and Kulozik [14].

Due to the poor deposit layer control in the inner channels of the SWM, the flux is reduced. The higher crossflow velocity in the outer parts of the module cannot compensate for that and the overall performance of the module is lower compared to the module with the diamond spacer. This is affirmed by the results on the protein permeation. It is shown in Figure 14 as a function of the Δp_TM_ at CF 1–3.

The discrepancy in protein permeation between the parallel and the diamond spacer becomes more intense with increasing CF, i.e., higher feed viscosity. At CF 1, the permeation of β-lg was almost similar with both spacers with 56% and 60% for the diamond and the parallel spacer (at a Δp_TM_ of 0.5 bar), respectively. At CF 3, however, the permeation of the parallel spacer was distinctly lower with 39% compared to 48% for the diamond spacer. Although the mean crossflow velocity at CF 3 was similar in both SWMs (compare Figure 12a), the crossflow velocity was significantly more inhomogeneous in the SWM with the parallel spacer due to the low channel height in the inner parts of the envelope. In consequence, deposition was more intense in the inner parts of the module, reducing protein permeation. Since the higher crossflow velocity at the outside could not compensate for this effect, the overall performance of the module is reduced. Therefore, a homogeneous crossflow velocity distribution in the radial direction seems to be crucial for the filtration performance of SWMs. Due to the differences in the crossflow velocity distribution, a potential additional effect of the feed viscosity on the local performance of the feed spacer could not be observed in the SWMs.

## 4. Conclusions

The results of this study showed that staining by CBB is an effective method to characterize the deposit layer with regard to its spatial distribution. Although the sensitivity of the staining method is limited at high amounts of deposited material, the staining method can be used to visualize the effect of the spacer design on the deposition and thus can be an indicator of the wall shear stress distribution. This can be used for the verification of CFD results. However, the pattern on the membrane and the amount of deposited protein did not correlate with the filtration performance of the SWM during milk protein fractionation. This could be attributed to the observation that the parallel spacer does not keep the inner flow channels of the SWM open. Thus, the deposit layer control in the inner parts of the SWM is poor. Additionally, the active membrane surface is reduced and compensates the possible advantages of the more homogeneous deposit layer formation of the parallel spacer in comparison to the diamond spacer.

Furthermore, our results confirmed that the curvature of the feed channel and its influence on the radial distribution of the crossflow velocity have to be considered in the assessment of spacers. Although both spacers had an equal filtration performance in the flat channel test cell, the performance of the diamond spacer was significantly better in the SWM. Apart from the turbulence promotion, the more homogeneous feed flow distribution in the radial direction of the SWM was positive to the performance during skim milk protein fractionation. In this regard, investing effort to induce a homogeneous feed flow distribution in SWMs seems to be beneficial to the module performance.

## Figures and Tables

**Table 1:** Geometrical properties of the SWM and the test cell.

	Length [m]	Active Membrane Area [m^2^]
Test cell	0.2	8.00 × 10^−3^
SWM	0.96	16.35

**Table 2:** Mean composition of the caseins and major whey proteins α-lactalbumin (α-la) and β-lactoglobulin (β-lg) of the UF-concentrate CF 3.

	Mean Value [g L^−1^]	Standard Deviation [g L^−1^]		Mean Value [g L^−1^]	Standard Deviation [g L^−1^]
κ-casein	12.21	1.05	α_S1_-casein	14.20	2.02
β-casein	41.46	2.26	α_S2_-casein	36.22	0.42
α-la	4.58	0.03	β-lg	12.96	0.33

## Supplementary Materials

The following are available online at https://www.mdpi.com/2077-0375/10/4/57/s1, Figure S1. Relative protein content in the guanidine buffer as a function of dissolving time for deposit layers in the range of 4 g m^−2^ to 24 g m^−2^. Error bars represent the standard deviation of two individual experiments; Figure S2. Absolute (a) and relative (b) composition of the feed and the deposit layer created by the filtration of skim milk (CF 3) with a diamond and a parallel spacer for an axial pressure drop of 1.0 bar m^−1^. Error bars represent the standard deviation of two individual experiments; Figure S3. Deposit layer formed by a parallel spacer (46 mil) after filtration of concentrated skim milk (CF 3) at an axial pressure drop of 1.0 bar m^−1^ (mean crossflow velocity of 0.14 m s^−1^). Flow direction from the right to the left; Figure S4. Deposit layer formed by a diamond shaped spacer after filtration of concentrated skim milk (CF 3) at an axial pressure drop of 1.0 bar m^−1^ (mean crossflow velocity of 0.21 m s^−1^). Flow direction from the right to the left; Figure S5. Cumulative distribution of the deposit layer pattern on the membrane for the parallel and the diamond spacer. The shaded area marks the 95 % confidence interval for the mean value derived from two individual experiments. Parts of the area are covered with more than 10 g m^−2^, which cannot be quantified by the staining method. Therefore, the cumulative distributions end below the value of 1.
