# Supplementary material for: Influence of Spacer Design and Module Geometry on the Filtration Performance during Skim Milk Microfiltration with Flat Sheet and Spiral-Wound Membranes
Source: Membranes (Basel). 2020 Mar 26;10(4):57. doi: 10.3390/membranes10040057 (PMC7231382; doi:10.3390/membranes10040057)
Supplement: Supplementary file 1 [file membranes-10-00057-s001.pdf]

membranes

Supplementary Materials: Influence of Spacer Design and Module Geometry on the Filtration Performance during Skim Milk Microfiltration with Flat Sheet and Spiral-Wound Membranes

Martin Hartinger *, Jonas Napiwotzki, Eva-Maria Schmid, Dominik Hoffmann, Franziska Kurz and Ulrich Kulozik

Chair of Food and Bioprocess Engineering, Technical University of Munich, 85354 Freising, Germany; jonas@napiwotzki.de (J.N.); ga96guz@mytum.de (E.M.S.); hoffmann.dominik1@gmx.de (D.H.); franziska.kurz@tum.de (F.K.); ulrich.kulozik@tum.de (U.K.)

* Correspondence: Martin.Hartinger@tum.de; Tel.: +49-8161-71-3719

Received: 15 February 2020; Accepted: 24 March 2020; Published: date

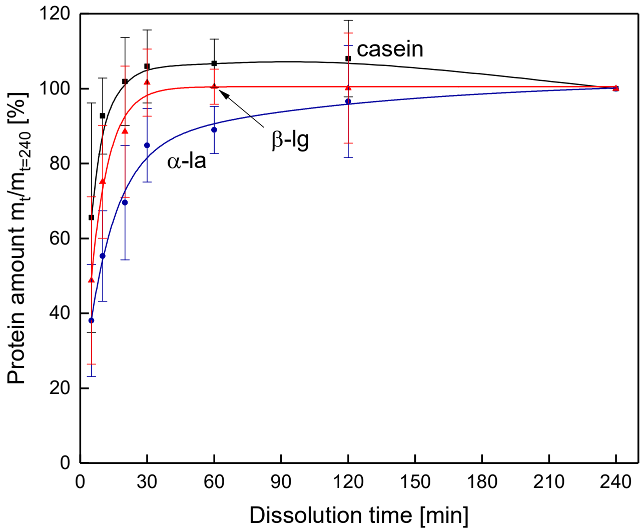

Figure S1. Relative protein content in the guanidine buffer as a function of dissolving time for deposit layers in the range of 4 g m^{-2} to 24 g m^{-2}. Error bars represent the standard deviation of two individual experiments.

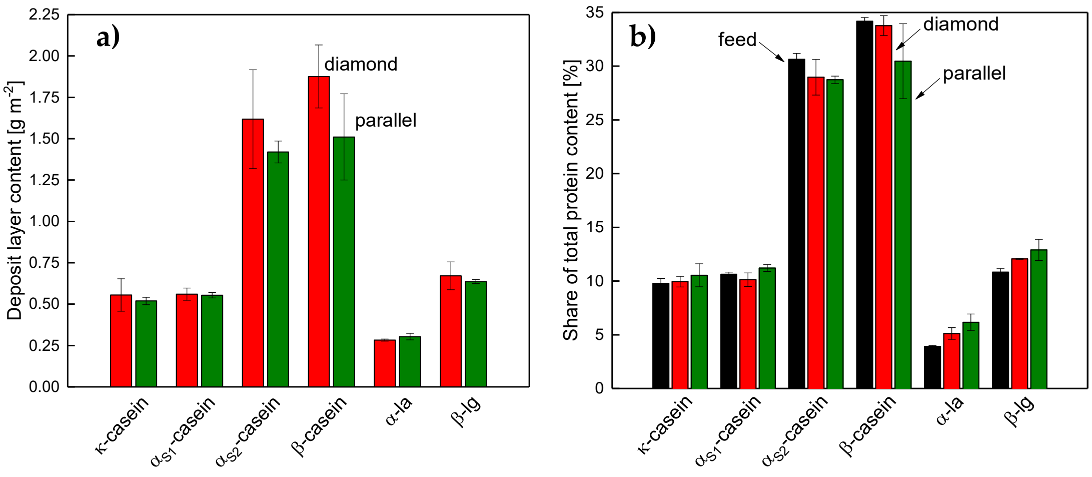

Figure S2. Absolute (a) and relative (b) composition of the feed and the deposit layer created by the filtration of skim milk (CF 3) with a diamond and a parallel spacer for an axial pressure drop of 1.0 bar m^{-1}. Error bars represent the standard deviation of two individual experiments.

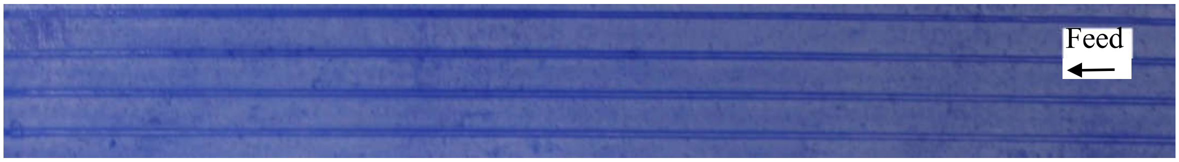

Figure S3. Deposit layer formed by a parallel spacer (46 mil) after filtration of concentrated skim milk (CF 3) at an axial pressure drop of 1.0 bar m^{-1} (mean crossflow velocity of 0.14 m s^{-1}). Flow direction from the right to the left.

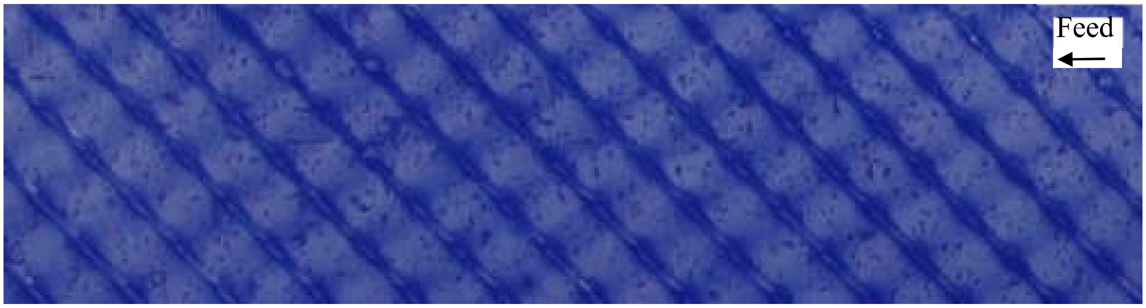

Figure S4. Deposit layer formed by a diamond shaped spacer after filtration of concentrated skim milk (CF 3) at an axial pressure drop of 1.0 bar m^{-1} (mean crossflow velocity of 0.21 m s^{-1}). Flow direction from the right to the left.

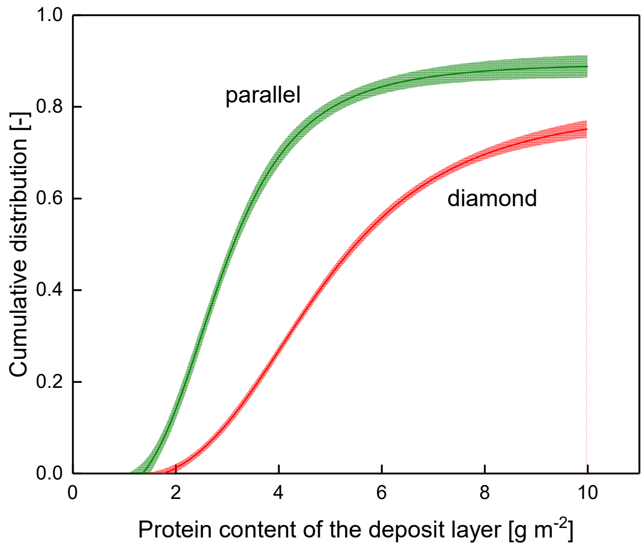

Figure S5. Cumulative distribution of the deposit layer pattern on the membrane for the parallel and the diamond spacer. The shaded area marks the 95 % confidence interval for the mean value derived from two individual experiments. Parts of the area are covered with more than 10 g m^{-2}, which cannot be quantified by the staining method. Therefore, the cumulative distributions end below the value of 1.

© 2020 by the authors. Submitted for possible open access publication under the terms and conditions of the Creative Commons Attribution (CC BY) license (http://creativecommons.org/licenses/by/4.0/).
